# Dataset for a Norwegian medium and low voltage power distribution system with industrial loads

**DOI:** 10.1016/j.dib.2023.109121

**Published:** 2023-04-05

**Authors:** Susanne Sandell, Daniel Bjerkehagen, Bjørn Birkeland, Iver Bakken Sperstad

**Affiliations:** a SINTEF Energy Research, Trondheim, Norway; b Norgesnett AS, Fredrikstad, Norway

**Keywords:** Grid data, Load data, Active distribution grid planning, Flexibility, Grid customers

## Abstract

This article presents a dataset for a Norwegian industrial medium voltage (MV) and low voltage (LV) electric power distribution grid with load time series. The raw dataset was collected in collaboration with the Norwegian distribution grid company (DSO^1^) Norgesnett as part of a pilot project in the Norwegian research centre CINELDI and was later anonymized and simplified into a secondary dataset, presented here. The load dataset comprises over three years of measurements of hourly load demand from 45 grid customers in the time period 2019-03-01 to 2022-03-16, collected from smart meters installed with the customers. The grid data comprises 2 radials of the distribution grid, collected from the network information system of the DSO. The data was anonymized and simplified. The dataset can be used for stochastic load modelling, analysing the grid's need for flexibility and power flow analyses for grid planning purposes.

**Specifications Table**

**Table:** 

Subject	Electrical and Electronic Engineering

Specific subject area	Active distribution grid planning and analysis
Type of data	Tables
How the data were acquired	The DSO provided the raw data. Grid data was acquired by combining information from different parts of the network information system (NetBas) and discussing with the DSO. The load data time series were hourly load demands measured from three-phase smart meters of the type OMNIPOWER (from Kamstrup AS, Denmark) installed with the end-users. The Norwegian Meteorological institute provide open access to temperature data from measuring stations on a CC BY 4.0 Licence. Data loading and preprocessing was done through a designated module in a custom-built Python code base, openly available from GitHub [1]
Data format	Secondary
Description of data collection	Raw grid and load data from a real industrial distribution grid was obtained from the DSO. The data was anonymized and the grid was simplified to generate a set of secondary data as described in detail in the Experimental design, materials and methods section.
Data source location	The raw data was collected by the DSO from the Øra industrial area outside Fredrikstad, Norway. The exact location of the grid assets is not given to preserve the anonymity of the grid users. Temperature data is collected from the Strømtangen Fyr meteorological station.
Data accessibility	Repository name: Dataset for a Norwegian medium and low voltage power distribution system with industrial loads Data identification number: 10.5281/zenodo.7763891 Direct URL to data: https://doi.org/10.5281/zenodo.7763891 (The raw data that the dataset presented here is based on are confidential.)
Related research article	*S. Sandell, D. Bjerkehagen, I.B. Sperstad, Load Analysis for Evaluating Flexibility Needs in the Planning of an Industrial Distribution Grid, International Conference on Smart Energy Systems and Technologies (SEST),5.-7. September 2022.* doi:10.1109/SEST53650.2022.9898467. [2]

## Value of the Data

• Although many grid models are available online, they are typically test grids that are not representing a real grid. The strength of the grid and load data described in this article is that they describe a real, Norwegian grid with both commercial and industry loads, with hourly load data collected over three years. The load data may represent typical Norwegian electric power usage, where space heating and thus temperature-dependent load makes up a significant amount of the total load. This data is useful for studying a Norwegian distribution system with industrial loads.
• The data can be used to analyze historic loads (aggregated or disaggregated), the load data can be used to model future load behavior, analyze the need for power system flexibility, and reliability of supply analysis. The data can also be used to study load patterns for different end users, such as commercial and industrial loads. The data can also be used as inputs to a grid planning case study, for instance using active measures (such as flexible resources) as well as grid investment measures as part of the grid planning.
• The data can be used for method development for evaluating remaining capacity in the distribution grid, in normal operation and in outage situations where customers on one radial must be supplied by a backup supply radial [3].

## 1 Objective

The data was initially gathered in the Norwegian research center CINELDI^2^ to study load modelling and to develop methodology for analyzing the need for flexibility services in an industrial grid area, which is described in a peer-reviewed conference paper [2]. The dataset is made public to facilitate others who wish to investigate methodology for planning of active distribution grids, or other distribution grid studies [4]. The data may be useful to Norwegian DSOs, the Norwegian transmission system operator (TSO), students or researchers and technology providers/innovators in the electric power distribution industry.

## 2 Data Description

The presented data describes a real Norwegian low-to-medium voltage distribution grid. The combined dataset comprises:

• **A grid dataset** that is a selection of the distribution grid pertaining to a substation (HV/MV)^3^ in Øra industrial area, more specifically, two radials in this area.
• **A load dataset** measured by smart meters at all the grid customers on the two mentioned radials, containing over three years (2019-03-01 to 2022-03-16) of load data with hourly resolution This data is described in detail in the following subsections.

### 2.1 Grid Data

**Fig. 1:**
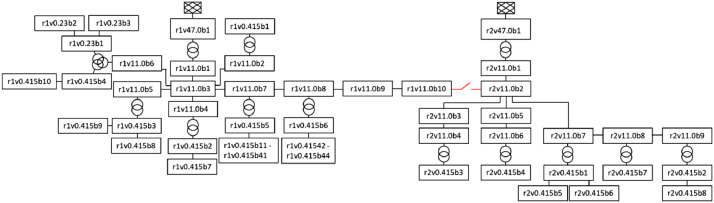
An illustration of the grid, including the names of all buses. Radial 1 (left) and radial 2 (right) are connected by one branch (colored red) which can be in service (when the switch (disconnector) is closed) or out of service (switch open).

Table 2 gives an overview of the files comprising the grid data. Fig. 1 is an illustration of the grid, including bus names. It is a MV and LV distribution grid comprising two radials of underground cables with a total of 76 buses and 75 branches. The voltage levels in the system are 47, 11, 0.415 and 0.23 kV. See Table 1 for an overview of how many buses are at each voltage level. There is only one load point per bus. One branch (colored red in Fig. 1) connects the two radials. The main feeders are located at bus r1v47.0b1 and r2v47.0b1,both at 47 kV. These two buses are the main feeders of their respective radials. Bus r1v11.0b10 and r2v11.0b2 are connected by a branch. This branch is a reserve (backup) connection between the two radials (depending whether it is *in service*, a term described in the following section).

**Table 1:** Number of buses at each voltage level.

Voltage level (kV)	Number of buses
47	2
11	19
0.415	52
0.230	3

### 2.2 Technical Specifications in Grid Data

**Fig. 2:**
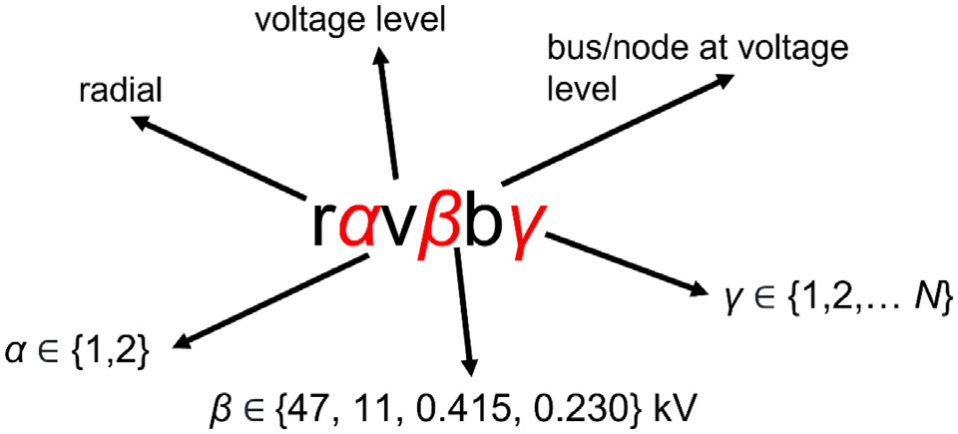
The naming convention of the buses in the grid, as automatized by the code base [1] in the data loading and preprocessing module.
• bus_type: bus type (1 = PQ, 2 = PV, 3 = ref, 4 = isolated)
• Pd: real power demand (MW)
• Qd: reactive power demand (MVAr)
• Gs: shunt conductance (MW demanded at V = 1.0 p.u.)
• Bs: shunt susceptance (MVAr injected at V = 1.0 p.u.)
• bus area: area number (positive integer)
• Vm: voltage magnitude (p.u.)
• Va: voltage angle (degrees)
• base_kV: base voltage (kV)
• zone: loss zone (positive integer)
• Vmax: maximum voltage magnitude (p.u.)
• Vmin: minimum voltage magnitude (p.u.)

The files described in Table 2 on the MATPOWER format contains various technical specifications. Table 3 shows an excerpt from *bus.csv*. Below, the different column names are described. The data columns in Table 3 are defined as follows (in accordance with [5]):

• bus_i: bus name (see naming convention in Fig. 2)

**Table 2:** Overview of files in the grid dataset.

File name	File description
bus.csv	Bus data on the MATPOWER case format [5]
branch.csv	Branch data on the MATPOWER case format [5]

**Table 3:** An excerpt from the file bus.csv.

bus i	bus type	Pd	Qd	Gs	Bs	bus area	Vm	Va	base KV	zone	Vmax	Vmin
r1v47.0b1	3	0	0	0	0	1	0	0	47	0	1.06	0.94
r1v11.0b1	1	0	0	0	0	1	0	0	11	0	1.06	0.94

Values in the real and reactive power demand columns (Pd and Qd) are all zero because when the data set is used, it is intended that values from the load demand time series will be used for these variables.

As can be seen in Table 3, the buses have unique names.^4^ They are automatically named in the data loading and preprocessing module in the code base [1]. The naming convention, illustrated by Fig. 2, is meant to give some additional information about the bus.

• ”*rα*”, tells which of the two radials the bus belongs to, ”r1” or ”r2”.
• ”*vβ*”, gives the voltage level (in kV) of the bus, such as ”v11” for 11 kV
• ”*bγ*”, is simply the number of the bus on that voltage level (b1, b2, ..., bN).

Table 4 shows an excerpt from *branch.csv*. Below, the different column names are described.^5^ The data columns in Table 4 are defined as follows (in accordance with [5]):

• f_bus: “from” bus number
• t_bus: “to” bus number
• br_r: resistance (p.u.), set to zero if unknown
• br_x: reactance (p.u.), set to zero if unknown
• br_b: total line charging susceptance (p.u.), set to zero if unknown
• rate_A: MVA rating A (long term rating, set to 1000 if unknown)
• rate_B: MVA rating B (short term rating, set to 1000 if unknown)
• rate_C: MVA rating C (emergency term rating, set to 1000 if unknown)
• tap: transformer off nominal turns ratio, (taps at “from” bus, impedance at “to” bus, i.e. if r = x = b =0, tap = $\frac{|V_{f}|}{|V_{t}|}$, where Vf and Vt are bus voltages at the from and to bus, respectively)
• shift: transformer phase shift angle (degrees), positive → delay
• br_status: initial branch status, 1 = in-service, 0 = out-of-service

**Table 4:** An excerpt from the file branch.csv.

f_bus	t_bus	br_r	br_x	br_b	rate_A	rate_B	rate_C	tap	shift	br_status
r1v47.0b1	r1v11.0b1	0	0	0	1000	1000	1000	1	0	1
r1v11.0b1	r1v11.0b3	0.0077	0.0064	0	7.62	7.62	7.62	0	0	1

The base value for calculating resistance [R] and reactance [X] per unit is:

$$Z=\;\frac{\text{basek}V^{2}}{\text{baseMVA}}=\;\frac{11^{2}}{10}=12.1\;\Omega$$

And the resistance and reactance [Ω] is found by multiplying the resistance (p.u.) by the base value:

R[Ω]=R[p.u.]×Z

X[Ω]=X[p.u.]×Z

### 2.3 Load Data

The load data associated with the grid presented in the previous section consist of time series load data with hourly resolution in the period 2019-03-01 to 2022-03-16. The load is measured in kW as an average load per hour. Similarly, it can also be interpreted as the total electric energy used that hour (given in kWh). The data is split among 45 files, named after the 45 buses in the grid with an associated load. See Table 5 for an overview of the files in the load dataset (Table 6).

**Table 5:** Overview of files in the load dataset.

File number	File name	Data description
1	r1v0.23b2.txt	Load data for bus r1v0.23b2
2	r1v0.23b3.txt	Load data for bus r1v0.23b3
45	r2v0.415b8.txt	Load data for bus r2v0.415b8

**Table 6:** Excerpt from a file in the load dataset.

Measuring point	Timestamp	Load (kWh)
r1v0.23b2	01/03/2019 05:00:00	203
r1v0.23b2	01/03/2019 06:00:00	249
r1v0.23b2	01/03/2019 07:00:00	370
r1v0.23b2	01/03/2019 08:00:00	419

The load data has been corrected for measuring artifacts due to daylight saving's time.

Some of the grid users have connected to the grid during the recorded period, so they do not have load data recorded for the whole three years.

## 3 Experimental Design, Materials and Methods

The grid and load data presented here is real data based on raw data from the DSO Norgesnett. Some simplifications were made to the raw data before publishing, and we call the published version the *secondary data*. Below, the data collection and processing is detailed, first for the grid data, and then for the load data.

**Grid data:** The secondary grid data presented here was put together through a combination of methods. The raw grid data was exported in .csv-format from NetBas, a GIS-based network information system used by the DSO. The raw grid data was interpreted through discussions with experts at the DSO. The DSO originally provided data describing a larger part of their distribution grid, consisting of several radials connected to the same substation. Two interconnected radials where chosen. The names of the radials, as well as the name of each bus, were anonymized according to the previously described naming convention (see Fig. 2).

*Load data:* The load data was recorded using three-phase smart meters of the type OMNIPOWER (from Kamstrup AS, Denmark) installed at each grid customer. The data was transferred to the local DSO and stored in their data base. Identifiable load data of the grid users is defined as sensitive information. To maintain anonymity of the grid users, an anonymization procedure was done on the load data. In the raw data, each grid user has a unique number which is attached to each load measurement. The code base [1] has a module which loads and preprocesses the raw data. In this module, raw load data file is first split up into one file for each grid user. Then, the ID number of each grid user is replaced with the anonymous identifier of the bus using the previously described naming convention (see Fig. 2). The key file, i.e. where the original identifier is coupled with the anonymous identifier, is only stored on a local machine according to the Data Management Plan agreed to between the DSO and the authors.

## Ethics Statements

Identifiable grid- and load data is considered to be sensitive information. However, the dataset has been anonymized as described in the previous section. The publication of the anonymized dataset is done with the DSO's consent, and is detailed in a data management plan between SINTEF Energy Research, the DSO (Norgesnett) and the Norwegian University of Science and Technology (NTNU).

## CRediT authorship contribution statement

**Susanne Sandell:** Data curation, Investigation, Software, Visualization, Writing – original draft. **Daniel Bjerkehagen:** Data curation, Software, Writing – review & editing. **Bjørn Birkeland:** Investigation, Resources, Writing – review & editing. **Iver Bakken Sperstad:** Conceptualization, Supervision, Writing – review & editing.

## Declaration of Competing Interest

The authors declare that they have no known competing financial interests or personal relationships that could have appeared to influence the work reported in this paper.

## Data Availability

Dataset for a Norwegian medium and low voltage power distribution system with industrial loads (Original data) (Zenodo).

## Footnotes

2 CINELDI (Centre for Intelligent Electricity Distribution) is a Norwegian Centre for Environmental-friendly Energy Research (FME), 2016–2024, www.cineldi.no.

4 Note that that the values in the *bus_i* are not according to the MATPOWER format [5] since they are string values and not integer values. Values in the columns *zone, bus area, Vm* and *Va* were not needed for the purposes that the dataset was generated for and have been set to default values.

5 Values in the columns in *branch.csv* to the right of the 11^th^ column (br_status) were not needed for the purposes that the dataset was generated for. Therefore, they have been set to default values and are not shown in the excerpt of *branch.csv* in Table 5.

## Footnotes

3 HV = High Voltage, MV = Medium Voltage, LV = Low Voltage.
